# Public perception and acceptance of CCUS: preliminary findings of a qualitative case study in Greece

**DOI:** 10.12688/openreseurope.16663.3

**Published:** 2024-07-24

**Authors:** Kostas Stavrianakis, Jacob Nielsen, Zoe Morrison

**Affiliations:** 1 ABERDEEN BUSINESS SCHOOL, Robert Gordon University, Aberdeen, Scotland, UK

**Keywords:** Carbon capture, Utilization, Storage, Climate change, Greece, acceptance, public, qualitative, case study

## Abstract

The development and implementation of carbon capture, utilisation and storage (CCUS) technologies plays an increasingly important part in European Union (EU) countries’ decarbonisation policies and strategies. Several studies have shown the important role social acceptance plays in determining the outcomes of CCUS projects and how social acceptance is shaped by the national and local contexts. Yet most studies on CCUS and social acceptance have focused on a few northern European countries despite the increasing numbers of CCUS projects across the European Union. This study seeks to help address this gap by conducting a case study on how local dynamics shaped people's acceptance and awareness of CCUS in two separate Greek communities. Based on semi-structured interviews with community members near a CCUS pilot plant, and a focus group with community members from a potential storage site, this single case study explores the factors and dynamics that shaped the participants’ perceptions of CCUS technologies. Our findings indicate that, despite the low level of awareness of CCUS technologies, participants could draw on their situated knowledge to identify potential drawbacks with their application. We identified scepticism regarding the adoption of new technologies and the organisations involved based on past experiences, and a notable lack of provision of technology and location-specific information as well as public engagement by the project consortium. Our recommendations for future projects and community engagement include the early involvement of the public in project development, location-based transparent information, appropriate channels to facilitate knowledge exchange, and educational initiatives to build communities' capability to influence projects.

## Introduction

Technology development and implementation play an increasing role in climate change policy and initiatives. Both the United Nations (UN) and the European Union (EU) have emphasized the use of technological advancements for reaching their climate goals. For example, the UN established the “Technology Mechanism” in 2010 with the scope of advancing climate technologies and transferring expertise, and large parts of the EU’s flagship research and innovation programme “Horizon 2020” support technological advancements in climate change technologies. Companies and corporations are in the “hunt” for decarbonisation as their products and activities often contribute to the increase of greenhouse gases, contributing to climate change. Alongside governmental organisations, the private sector emphasizes the deployment of technological advancements to reduce both their environmental and social impacts ( Bokka & Lau, 2023; Krzywdzinski, 2019; Seemungal *et al.*, 2021).

Carbon capture, utilisation and storage (CCUS) has received increased support as a decarbonisation solution and climate change mitigation technology. Main greenhouse gas emitters such as the UK, EU, US, and China have put in place a range of policies and schemes that support the development and implementation of CCUS in order to meet their climate targets ( Friedmann *et al.*, 2020; Matschoss & Repo, 2018; Yang *et al.*, 2019; Zhang *et al.*, 2016).

In the debates around whether and how to develop and implement CCUS much of the focus has been on the role that scientists, organisations and governments play ( Bowen, 2011; Haszeldine, 2009; Mace *et al.*, 2007). However, it is increasingly recognised that it is necessary to take account of social acceptance through the implementation of relevant policies. The focus on social acceptance is partly due to how projects in the past have been cancelled due to community resistance, but it also stems from a concern with preventing adverse outcomes for local communities ( Anderson *et al.*, 2012; Reiner *et al.*, 2006; Shackley *et al.*, 2009; Wong-Parodi & Ray, 2009). Scholars have therefore pointed out that to ensure just and effective outcomes of climate change initiatives it is necessary to understand the dynamics that inform public acceptance and awareness ( Upham *et al.*, 2022; Williams *et al.*, 2021). 

Social acceptance of new technologies is deeply embedded in social dynamics and influenced by multi-dimensional political, educational, social, economic, cultural, and historical factors. To understand social acceptance, it is therefore necessary to examine it in relation to a complex and changing local context ( Bertsch *et al.*, 2016; Krupnik *et al.*, 2022; Sovacool & Ratan, 2012; Wüstenhagen *et al.*, 2007), whilst also recognising that local contexts are shaped by wider national and global dynamics ( Burawoy *et al.*, 2000). Despite this, most previous research on social acceptance is based on studies from a limited number of countries mainly located in Northern Europe or North America ( Nielsen *et al.*, 2022). This is perhaps not surprising given that many of the initial CCUS projects took place in these regions, however, there are increasingly new CCUS projects being implemented in other parts of the world, including in many EU member states in South Europe. Given the importance of the local context and how that context is shaped by national and global factors, it is therefore necessary to examine wider sets of experiences to gain a broader and more fine-grained understanding of the dynamics that shape social acceptance of CCUS projects.

Furthermore, it is important to consider the specifics of the technologies and how generic terms like “wind energy” or “CCUS” refer to clusters of diverse technological constellations, constituent parts of which may have different implications for social acceptance ( Hmielowski *et al.*, 2019; Lin *et al.*, 2007). Most research on CCUS has so far focused on the social acceptance of specific storage technologies, perhaps because much of the initial resistance to CCUS was centred around storage sites. However, CCUS includes a range of different technologies with different environmental and social implications that could each create different sets of perceptions and reactions from the public. It should not be assumed that resistance to CCUS will only occur in relation to one aspect of the technologies. As mentioned above, CCUS is not one single technology and process, but it is an ecosystem of processes and different technological innovations, that need to be studied in tandem rather than in isolation. Thus, this study is looking both at a carbon capture location, as well as a suggested carbon storage location.

To address the above gap concerning the different aspects of CCUS technologies in a location where the social dimensions of CCUS have been inadequately studied, this study explores public perceptions and awareness of CCUS technologies within a Greek context, focusing on two rural areas where in one of them a pilot project has been scheduled to operate and with the second area being a potential storage location for carbon dioxide. The pilot plant will be adjacent to an industrial plant that operates in the area, and the goal of the pilot is to capture part of the carbon dioxide emitted by the industrial plant. The potential storage area is located on an island approximately 250 kilometres away from the pilot plant. Despite the pilot plant only capturing carbon dioxide, the project explores the different societal, environmental, and economical dimensions of all parts of the CCUS technologies.

Greece is one of the EU member countries that has received little attention regarding public perceptions and CCUS. Anecdotical evidence suggests that, since the early 2000s, universities and research institutes in Greece have been experimenting with the development of carbon capture and storage (CCS) technologies through EU-funded projects. This may add to confusion locally as terms for CCS and CCUS are often used interchangeably with little explanation. We have identified only a limited understanding of public perceptions and social acceptance of CCS/CCUS in Greece. Pietzner *et al.* (2011), Koukouzas *et al.* (2022) and Sprenkeling *et al.* (2022) have looked at Greece in tandem with other EU citizens, but not as a distinct entity and not in relation to a specific project. There have been studies on social acceptance of technologies such as windfarms and solar panels in Greece ( Botetzagias *et al.*, 2015; Kontogianni *et al.*, 2014; Stigka *et al.*, 2014; Tsantopoulos *et al.*, 2014). However, CCUS technologies are often used in different contexts and can be integrated with hard to abate industries. This also means that to understand the dynamics that shape social acceptance of CCUS in Greece it is necessary to examine the specifics of these technologies.

Greece, therefore, makes an important case study for understanding the relationship between the implementation and development of CCUS and social acceptance. For one, given how CCUS are likely to play an increasing role in the Greek decarbonisation strategy it is important to understand the dynamics that shape local Greek social acceptance in relation to CCUS projects. Furthermore, by looking at case studies from understudied areas like Greece we can broaden our general understanding of how local and national dynamics shape social acceptance. Finally, by examining a specific CCUS project across the potential storage and capture site the paper can contribute to our understanding of the local dynamics that shape social acceptance of projects and how there might be similarities and differences across different sites impacted by the project.

## Methods

### Research design

To get a richer understanding of the phenomenon of CCUS perceptions we followed a single case study design ( Creswell, 2013; Yin, 2009) focusing on community experiences, knowledge, and acceptance of CCUS. A single case study design was chosen as it satisfies Yin's (2009) five rationales; 1) theoretical perspective, 2) extreme cases, 3) capturing a revelatory case, 4) access to a previously inaccessible case, and 5) the longitudinal nature of the case. In more detail, this embedded single case study explores the phenomenon of social perceptions and acceptance towards CCUS technologies in two rural Greek communities ( Yin, 2009). As this study is part of a bigger project that aims to inform the European Union on CCUS policy matters, we have employed an instrumental approach to our case study as our inquiry is based on that final product of policy writing ( Stake, 1995). We do not seek to generalize to the wider public, but to have a better understanding of the specific subunits we engaged with ( Stake, 1995; Yin, 2009).

### Case context-phenomenon

The phenomenon explored in this case study is the role of CCUS technologies in addressing industrial emitted carbon dioxide (CO _2_) in two rural areas in Greece whose economic activities rely on the extraction of natural resources, the tourism industry as well as agriculture. As with all case studies, it is important to define the boundaries of the case study including the sources of data and defining the unit of analysis ( Merriam & Tisdell, 2015).

The spatial boundaries where the phenomenon takes place are two settlements in Greece and surrounding villages.

The first settlement is in an area where one of the project’s industrial partners operates, and a pilot CCUS project will be installed on their premises for demonstrative purposes. The purpose of the pilot project is to demonstrate the capture process of CO _2_ from the company’s flue gas by testing the project’s innovative technologies. Once captured the CO _2_ will be released back into the atmosphere, as there are no plans to storage or transport the captured CO _2_ at this point of the project. The pseudonym of “ *Schatz GMBH”* has been given to the industrial partner for this study. It is important to note that the area and the regional residents’ living there, have been previously disrupted by the operations of an ore mining company, but not the mining company of this project. In the past regional and national protests have taken place to show the public’s opposition towards the company’s operations due to health and environmental degradation concerns in relation to extraction techniques and natural resources exploitation. For the purposes of this paper, this company is identified as “Ore *Extraction Limited*”.

The industrial partner’s commercial activities are in the mining industry and the development of products for use in industries such as iron and steel, nutrition, industrial and manufacturing, as well as mining and metallurgy. The company has been active in the area for more than five decades with a continuous presence in the mining history of the area.

Both the factory and the mining operations of the company are located in a settlement that did not exist prior to the mining activities. The settlement was built around those activities with workers moving from nearby villages closer to the factory by building houses leading to the development of this settlement. Today the local community is well integrated with the mining activities as most of the people living in the area are employed in this facility.

The company takes pride in their sustainability and societal initiatives for which they have received multiple awards and participated in several EU-funded sustainability projects. The current project that the company is a partner of, is another example of the initiatives that the company has taken to promote more environmentally friendly actions within their operations.

A substantial percentage of the company’s carbon emissions is a result of the kiln operations that are an essential part of its manufacturing process. For the company to be compliant with EU and national regulations and directives, including the European Union Emission Trading Scheme ^1^ (EU ETS), it needs to find a solution to decrease its carbon emissions.

The second settlement is located on an island of the Northern Aegean in close proximity to the Greek mainland. Oil and gas reservoirs are located within the coastal areas of the island and they have been exploited since the 1980s by different companies throughout the years. While some of the reservoirs are still active, others have been depleted and have been identified as suitable storage sites for carbon dioxide to reduce greenhouse gases { Avraam & Vatalis, 2023 #17502}. A recent report from the company that has the oil and gas exploitation rights in the area showed that the area can store up to 3 million tons per annum of carbon dioxide contributing to local and regional decarbonisation efforts { Energean, 2023 #17503}. The exploitation of the oil and gas in the area has received both support and opposition throughout the years { Stergiou, 2022 #17504}. 

Although there is no project partner associated with the storage site, and storage will only be modelled in this project, as researchers we chose to include community members nearby storage facilities to elicit their opinions and attitudes towards CCUS. 

### Data collection and analysis

When conducting research in a social setting that the researchers are not members of, it is important that the researcher gains the social acceptance of the community and identifies him/her-self ( Gillham, 2000). 

To this end, one of the authors (KS) spent an extended amount of time in the mainland community and throughout this time he established connections and befriended members of the local community. Despite the researcher not being from the area, he had knowledge of prior resistance to industrial activities in the area due to environmental and health concerns associated with the extraction of gold. He is a native speaker of the Greek language, and that allowed him to approach individuals who were more confident discussing CCUS matters in the Greek language, as well as pick nuances in the discussions that were linguistically particular to the Greek language.

Due to time constraints, to get in touch with community members of the island (islanders), KS made initial contact through a social media platform of a local organisation that is active in the cultural, environmental and tourism aspects of the island. After the initial contact through the social media platform, the communication continued through a series of emails. KS was in communication with a member of the organisation who was also the administrator of the organisation’s social media platform. KS had previously worked on the island and was familiar with the location and some of its cultural and environmental dimensions. 

Below we discuss in more detail the semi-structured interviews conducted with the mainland community members as well as the focus group we did with the community members on the island.

### Semi-structured interviews

To gain the necessary in-depth description of the phenomenon a series of semi-structured interviews were conducted with members of the local mainland community ( Creswell & Clark, 2017; Gillham, 2000). We followed a convenience sampling approach within the spatial boundaries of this case study. We used a semi-ethnographic approach in an effort to immerse ourselves in the community. Having prior knowledge that members of that local community had been on the frontlines of a controversial environmental related topic with a nearby mining operation, (not this project’s industrial partner), it was important to build rapport with community members to allow for insightful discussions and local knowledge sharing. Living within the community boundaries allowed the researcher to briefly embed himself in the daily life of the town. As an example, the author KS joined a local sports club where he had the opportunity to both inform locals about the project, but also gain important insights from informal discussions. In addition, it is difficult and often inconvenient for people to be available when the researchers want, thus living within the community boundaries allows for more flexibility in meeting people. Not discussed in the study, but the author KS, also collaborated with the local school, again in an effort to immerse himself in the local society and learn more from them. Participants were recruited through informal discussions and putting up posters for an upcoming community event in relation to this EU-funded project. Data collection spanned from November 2021 to January 2023. During this time the study site was visited three times and interviews were conducted at different times. The purpose of the interviews was to gain an understanding of participants’ awareness of CCUS technologies and how they perceived their application in their local context. As this is a case study, the scope of the participants’ insights is not to generalize their perspectives to the wider society, but to rather explore their own views and experiences with the proposed Carbon Capture facility ( Hammersley *et al.*, 2000 #17449). The purpose of the interviews is to explore why participants have certain opinions and perspectives about the pilot CCUS facility and how those opinions are shaped ( Yin, 2009 #1707( Cobern & Adams, 2020 #17450)9). Explorative studies with a low number of participants are important, as they can inform further research by building knowledge on less researched topics { Crouch & McKenzie, 2006 #15677}. In their paper, { Hennink *et al.*, 2017 #15678} have suggested that the main themes of a study can be identified within the first six conducted interviews, whereas similarly { Young & Casey, 2019 #15679} have suggested that the vast majority of themes and codes were evident in studies with a minimum of six interviews.

Prior to conducting the interviews participants were provided with a study information sheet, explaining the purpose of the study and their role as participants. Additionally, a consent form (both Greek and English versions were available) was provided to the participants to sign confirming their participation and comprehension of the ethical dimensions of their participation. This study received ethical approval from the Aberdeen Business School Research Ethics Committee at Robert Gordon University on the 10 ^th^ May 2022.

To initiate the discussion, a 3-minute video ^2^ was shown to the participants. The video was produced by the communication team of this CCUS project as a communication tool to the wider public, and it was the main mode of communication about the project.

The content of the video covered the aims of this current CCUS project as well as the promotion of CCUS as a decarbonisation strategy to reach net zero. The input of this study’s researchers to the video was minimal. To supplement the video the researcher provided some extra context in relation to potentially important issues such as CO _2_ transport and usage of resources such as water and energy.

In total five interviews with six members of the public were conducted exploring the role of CCUS as a decarbonisation strategy and the installation of such technologies in their area. All interviews were conducted by the author KS. The interviews had an average duration of 45 minutes and were audio-recorded and later transcribed verbatim. Four of the interviews conducted were one-to-one interviews and one was a dyadic interview ( Polak & Green, 2016). Each individual was interviewed only once. 

As four of the interviews were conducted in Greek and not all members of the research team spoke and comprehended the Greek language, it was essential that the interviews were translated into English for the analysis. The translation was done by one of the researchers, KS, who is bilingual in both English and Greek. Both Fersch (2013) and Filep (2009) have discussed the complications and necessary strategies in regard to translating interviews for analysis. We followed their recommendations for translating proverbs ( Filep, 2009) and considered the implications of our translation in the analysis of the interviews ( Fersch, 2013).

The translated transcription text was entered into a qualitative data analysis software, and thematic analysis followed. To conduct the thematic analysis, we followed Braun and Clarke's (2012) six-phase approach including data familiarisation, generation of initial coding, themes identification, themes review, theme naming, and report production. The coding was done both inductively and deductively considering both the data derived from the interviews, as well as from pertinent literature. The first author did the initial coding which was later refined with the input of the other two authors. ( Burrows & Kendall, 1997).

### Focus group discussion

Due to time constraints and limited participants’ availability, a focus group approach was deemed appropriate to elicit opinions and experiences from the community on the island (islanders){ Morgan, 2002 #17510{ Vanderstoep & Johnston, 2009 #17511}}. In continuation to the email exchanges mentioned above, a face-to-face meeting was scheduled together with other members of the organisation. The two researchers (KS and JN) travelled to the island to conduct the focus group session. The meeting’s arrangements i.e., date, time, location, and discussion topics were mutually agreed upon between the researchers and the administrator of the organisation who was in contact with other members of the organisation ensuring their participation. 

Similarly to the interview protocol, prior to conducting the focus group, participants were provided with this study’s information sheet, explaining the purpose of the study and their role as participants, as well as the consent forms. As some participants preferred not to audio record the conversation, no audio recordings took place during the focus group; instead, the researchers kept field notes of the discussions. 

As all participants were native speakers of the Greek language and so was one of the researchers, the focus group was conducted in the Greek language. The focus group consisted by five participants. Two more residents were expected, but due to other responsibilities they could not make it on the day.

The focus group was hosted in the administrator’s house as all participants were familiar with it, and it was the easiest option from a logistics perspective. We gathered in a room, and as soon as all participants arrived, we started the focus group discussion. An initial PowerPoint introduction was given to the participants, and similarly to the interview protocol the project’s video was shared with the participants as an engagement tool. The duration of the focus group was approximately two hours, but the conversation continued after the focus group had finished and we moved to the garden of the house for a refreshment.

As the focus group discussion was not audio-recorded, the results section below is based on the field notes that both researchers collected throughout the day. Both researchers kept short notes during the focus group discussion, and as soon as possible they developed more comprehensive field notes based on the short notes { Phillippi & Lauderdale, 2018 #17512}. 

During the discussion the researcher leading the focus group, made it clear that no carbon dioxide storage would take place as part of the project, and that it was just the processes of capture and utilisation that would take place.

## Results

As the communities where this case study took place are small and to protect the participants’ identities a minimal number of participants’ demographics will be shared.

For the mainland participants three were female and three were male. They were all residents in the area working in different sectors of the local economy e.g. education, food and beverage, agriculture, tourism, etc. Their ages varied from the mid-thirties to late forties. Individual participants will be distinguished below with their pseudonym initials in brackets, i.e. [T] for Tim, [A] for Alex, [P] for Peter, [J] for John, [O] for Olga and [M] for Mariah.

Of the island participants, four were male and one was female with all of them being residents of the island. Participants had diverse employment backgrounds, with some being employed in the tourism industry, others being pensioners, and others being politically active on the island. As above, participants will be distinguished by their pseudonyms, i.e. Jerry [JE], Charles [C], Bill [B], Sarah [S], and Reno [R].

Below we present the three themes identified in our data, namely: Knowledge and Concerns, Societal Context, and Technology. It is important to note that although the themes are presented below on an individual basis, the intersections amongst them are the novelty of the paper. As an example, knowledge and concerns cannot be isolated from previous experiences which is part of the societal context theme. 

A summary of the results is provided in Table 1, and a more detailed comparative presentation of similarities and differences between the two sites will follow.

**Table 1.** Summary of results.

Theme	Mainland Carbon Capture Site	Island Storage Site
Knowledge and Concerns	● Low prior awareness of CCUS. ● Ability to use their knowledge to explore the impacts of CCUS. Shared Concerns: ● Risks associated with carbon dioxide leakage. ● Global and local implications. Unique Concerns: ● Natural Resource Usage. ● Electrical Power Usage. ● Tourism. ● Health issues. ● Transport Infrastructure. ● Environmental Pollution (amines).	● Low prior awareness of CCUS. ● Ability to use their knowledge to explore the impacts of CCUS. Shared Concerns: ● Risks associated with carbon dioxide leakage. ● Global and local implications.
Societal Context	Shared: ● Lack of trust in companies due to previous experiences of conflict. ● Ambivalent relationship with the EU. ● Lack of transparency. ● Doubt about the distribution of the economic risks and benefits from CCUS technologies.	Shared: ● Lack of trust in companies due to previous experiences of conflict. ● Ambivalent relationship with the EU. ● Lack of transparency. ● Doubt about the distribution of the economic risks and benefits from CCUS technologies. Unique Concerns: ● Lack of trust in local politicians
Technology	Shared: ● Trust in the potential of CCUS technologies to address climate change. ● Sceptical of oversimplified communication about CCUS technologies. ● Required more information to understand the different implications of CCUS technologies. Unique Concerns: ● Uncertainty on how is responsible for communicating information about the project.,	Shared: ● Trust in the potential of CCUS technologies to address climate change. ● Sceptical of oversimplified communication about CCUS technologies. ● Required more information to understand the different implications of CCUS technologies.

### Theme 1: Knowledge and concerns

Prior to showing the project video, most participants had little to no exposure or awareness of CCUS technologies. One of the interviewed participants said:

[O]: “ *I didn't know anything before I saw this particular video and I hadn't heard of it [CCUS] before, I just heard about it at some point, it may be that I saw an advertisement on TV about it*”.
All focus group participants but one, had also little to no knowledge of CCUS. One of the focus group participants [C] was very familiar with CCUS as he had previously worked in the local oil and gas industry and has an advanced university degree in the natural sciences.

Once provided with some basic information about CCUS through the video, participants were able to draw on their knowledge to critically examine and identify the local implications the technology could have on natural resources, agriculture, tourism, health, and transport.

There were some concerns about the use of natural resources in connection with the implementation of CCUS, particularly the existential need for water and in terms of supporting the agricultural industry that relied on access to water resources. That concern was mainly voiced by participants located on the mainland.

[T]: “For *the mines that already using water for the mining activity. So, we'll need extra water in order to separate this thing. That means more resources for the mines water, uh, resources of the region will be used… sorry, but the water, what somebody water is the basic, uh, biological need for life. I mean, is the, the, the, the number one thing that people should be concerned in this area to have enough water. Because there is also another mining activity in the west, uh, of the region, Woodend that is, uh, has to do with ores, and on this area that they're doing, the mining activity is practically the resources for the whole water resources”*.

[J]: “ *Water in principle is an issue for all of us in the coming years everywhere. If we take our own region, Woodend, but also by extension and as a reference point that we are talking about Timberville, it is a big gamble in the coming years. Why? Because the edible olive ripens at the very time when we have tourism in the respective region and the corresponding arrivals and visitor numbers. If we would need 1 litre of water when we had no tourists or vice versa for a tree I say it randomly, now we need 3”.*
Despite the islanders not having experiences with CCUS, they drew their concerns on the socioeconomic and environmental dimensions of CCUS from past experiences with both mining and oil and gas companies operating locally. As an example, participants were concerned with the risk of carbon dioxide escaping when stored in the seabed. To that [C] said that there is no such risk as the top is sealed. Although participants trusted [C], that same concern was mentioned multiple times throughout the discussion.

Similarly to the above concern, the same issue was raised by a mainland participant where storage is not going to take place.

T]: “ *Are we sure that this, uh, CO _2_ under sea or underneath on the earth is not damaging the microcosmos of the underground? And because we don't see it on the air, we think we solve the problem. It's like the problems we put under the carpet. Do we know that the microorganisms who live inside there, and I'm sure there are, uh, are not, um, affected by all this? Do we know it? What have we put to see what happens inside there? Did we count the microorganisms inside the caves before and after? Do we know that? Or we just save the atmosphere, and we messed up the earth?”*.

To a somewhat lesser extent some of the mainland participants raised the issue of where the electricity to power the carbon capture facilities would come from was raised. That was not a topic discussed by the islanders.

[A]: “ *Do we have enough solar energy in the area to [power the plant]? … It said [video] that's gonna be powered by renewable energy.”*

For context, the region has already experienced an increase in solar energy that has displaced some agricultural activities.

Tourism is an important source of employment and income generation for both areas, but only the mainland participants expressed their concerns about CCUS and tourism.

[J]: “ *Of course, and for the tourists that's a concern, but we also have the primary production which is also very close by. As it is a tourist activity, you see hotels at a distance from the flue gas with an example of Timberville and “GMBH”, the distance to the nearby hotel is 500 meters in a straight line. It may be 2 km, rather Guest House in a straight line is 1 km, so next-door. And an olive tree grown edible is one meter <laughs ironically*>”.

Potential health issues were also discussed by the mainland participants regarding the use of amines for the capturing process. Despite not using amines in this project, amines are one of the most common absorbers to use in the capturing process and might have potential health impacts ( Gentry *et al.*, 2014). Despite the researcher making the same point about amines to both the mainland community and the islanders, only participants from the mainland community chose to expand on that point.

[R]: “ *Another issue with carbon sequestration is when carbon dioxide is released and we want to capture it, we use chemicals*”.
[O]: “ *A*” (okay)
[R]: “ *So, its what chemicals do we use? Because in the past, for example, they used what they call amines and amines can be carcinogenic*”.
[O]: “ *So, these are going into the environment? Or to the employees*?”

As a rural area with somewhat limited transport infrastructure, there were also concerns that the implementation of CCUS could cause issues. That concern was voiced by the mainland community and not by the islanders.

[T]: “… *personally, I would like to see some numbers in the sense the whole process is what we were also saying the other day, the whole process …the part of the transportation*. *Do we have any idea, sorry, uh, how many trucks we will be leaving daily from the place in order to go? Because Woodend doesn't have really roads, big roads, it’s very small roads, it's one lane, one lane each, old roads”.*

The participants also critically identified and analysed issues that were not local. For example, one participant questioned the premise that some industrial CO2 emissions were unavoidable, whereas another participant from the island had questions on the source and amount of CO _2_.

[A]: “ *That it's saying, the problem is that for some industrial activities, we cannot avoid producing CO2. Not even, we cannot avoid reducing the productions. We cannot avoid the production of CO2. So, we are trying to solve this problem because we are basing the whole idea that this is something that we can’t change. Why guys? Is there that we need to do research?*”.
[JE]from the focus group brought up the issue of scale, and questioned whether the carbon dioxide emissions in Greece can justify such an investment and scale, or whether carbon dioxide from other countries would also be deposited in their area. In extension to this question, [B] expressed his concern that if Greece does not produce enough CO2, and more needs to be imported by other EU countries, then their area around the island would becoming a dumping ground for the Europeans.

[Participants enhanced the conversation with their local knowledge and cultural perspectives that are important elements in public engagement and knowledge production.

### Theme 2: Societal context

Participants' perceptions of the CCUS project were understood in relation to past experiences and perceptions of companies and political institutions. Most participants voiced concerns related to issues of transparency, trust, and political-economic dynamics.

In both interviews and the focus group discussion, the company “ *Ore Extraction Limited*” whose activities some years before had led to prolonged protests and conflict was mentioned in some capacity or another. The way the conflicts were handled by the company and the local and national politicians meant that there were concerns about how new initiatives could be handled.

[P]: “… *there was also deception, meaning that they were telling us that the mining process here had been used previously in other places and that was not true. It had only used in preliminary studies and pilot projects, meaning no proper mining was done like that and it will never be done, it is not possible. The production cost of this process is too high, and they eventually turned into open cast mining, like we are living in the 1800s*”.
[R] who is employed in the tourism industry, also mentioned the “Ore Extraction Limited” and brough their activities as a reason to have little faith in companies and their ethics. To that, [JE] suggested that the oil and gas company might already deposit carbon dioxide without them knowing, as they do have the exploitation rights of the area, and nobody can tell them otherwise. [R] suggested that if they wanted to change something and have their voices heard, they should actively protest as they did in the past with the installation of military radars on the island.
[B], who is politically active on the island, said that recently all the decisions taken had a financial weighting rather than a political and he was concerned the same would happen with CCUS. To expand this point, [JE] mentioned that the oil and gas company does not employ Greeks at their rigs, because they do not have the expertise. Instead, they employ workers from abroad who work for less money.

This in turn led to a hesitant stance on any new initiatives even those that might turn out to be a good thing for the area.

[P]: “… *Of course, I will agree that social acceptance is needed in anything that is new because there is the bad from the past. Meaning that people are cautious in anything that happens, and many times they are cautious in good things”.*

The relationship with the local companies that would potentially utilise CCUS technologies was also complex. On the one hand, the jobs they created were seen as being integral to the local community, and on the other hand, in the past there had been issues with pollution and the decisions not to act up on it, as one participant described.

[T]: *“I see around also my family back in the days used to work for the mines, Okay? That families living from the mines, uh, local, uh, community, most of them, let's say, because it's a small village, work in the mine. And I see that some things that perhaps mining activity is uh, uh, uh, um, getting lower the quality of life, like the dust that we breathe every day from the mines, I see that people put it on the scale and they say, okay, but we have work.”*
Based on their previous experiences, the islanders were very apprehensive of the oil and gas company that has the exploitation rights and were interested to know what the benefits to the local community would be from storing CO _2_ in the area.

The ambivalent relationship with the EU was brought up by both mainland and island participants. On the one hand, there was the perception that the EU was lacking transparency and supported the interest of the companies at the cost of the environment and society. On the other hand, the EU was also seen to have the potential to ensure social and climate-beneficial outcomes when these new technologies.

[O]: *“…if it's to serve the interests of the industries, which it (EU) potentially does in many cases because lobbying and behind the scenes is everywhere. So, in this case and the way you present it, it seems that the idea of the European Union is what touches the citizens and works for society. So, in that sense it is clear research-wise that these approaches, these storage options are worth to invest money, I now take it differently. Now coming from you, since the European Union is funding these kinds of proposals, is it clear that these options do not degrade the environment?... it should be, because I am thinking about it for the citizens and for their good, but at the same time I am thinking about it. Am I (EU) funding proposals that offend and degrade the environment in which the societies, the European ones, live. There is a bit of an inaccuracy here*”.
Focus group participants discussed the role of the EU, but not to the extent that interview participants did. The main discussion was about the use of EU funds to develop technologies that would promote the interests of private companies.

For some, the EU was seen as important in creating the legislative pressures to push companies to decarbonise.

[J]: “ *Because without having, having probably 10–20 years ahead or whatever it is in exploitation, they want at all costs that this is profitable and for it to be profitable they have to be okay with the legislation. They have to comply with what the national and possibly European legislation dictates*”.

Despite the potential benefits of addressing climate change that EU support for CCUS could have, there was some scepticism about the lack of transparency, especially in terms of who would receive the financial benefits from these projects.

[T]: “ *What the European citizen wins, uh, at the end of all this?”*
[A]: “ *Take the CO2*”.
[T]: “ *So cleaner air*?”
[A]: “ *And this project is financed by*...”
[T]: “ *Yeah, but some other people will get (money) <laugh>. That's the funny part. You know, we, we pay some others are gonna be profitable. And with this data, with this lack of data, let's say, and all this process that it has vague, points, I would say that is another, I don't, um, I don't see it in a good, uh, way after all, if I start looking all the details, where they're gonna go, who's gonna take the money? Who, who's gonna do this?*”

In the above statements the participants demonstrated some of their experiences with industrial partners, while expressing their opinions on the role of the EU in decarbonisation.

### Theme 3: Technology

Most participants believed that technologies and potentially CCUS could play a role in addressing climate change. They were however critical of the little information they had received about the technologies used in the local CCUS project. To form a proper opinion on the specific technologies used in the project they expressed the need for better communication and education. Interestingly, most participants had a positive attitude towards technologies like CCUS addressing climate change. One participant who was involved with the local commons discussed how the research advances and societal changes have contributed to technology being an important element. The islanders in particular placed a lot of trust to Charles, as the expert, when they had questions and uncertainties on the technological aspect of CCUS and more specifically the storage of CO _2_.

[J]: “ *I think the way our society has formed, and the way research has evolved in general, I think technology is a key element that can address these kinds of problems, … I think it is impossible that something can happen without the intervention of any technological (applications). As the video shows, you are producing this dioxide from an industrial facility, showing that you can have underground storage for example, all of that again gives you the opportunity in finding the solution, but again having a storage, randomly I will say, either above ground or underwater without technology again you can't actually cope*”.

Another participant discussed the importance of scalability, as well as the complementary role that nature-based solutions and technological innovations should have.

[M]: “ *Yes, I think they are complementary (nature and technology), but I think if a technological solution is found it will be more effective, just because of the scale… I* “ *Yes, I think they are complementary (nature and technology), but I think if a technological solution is found it will be more effective, just because of the scale… I think technology can always play a very positive role, as long as it's used properly*”. *think technology can always play a very positive role, as long as it's used properly*”.

Despite this receptivity to technology and generally positive attitude to the potential of CCUS, there was some criticism about the project video.

After the interview, some of the participants were sceptical about the video. The video was described as propaganda, as they thought it oversimplified the CCUS process and was lacking important information. Another participant said that the video did a very good job explaining the CCUS process, but that it could be confusing as important information was not mentioned.

[J]: *“This video is very good as far as I would say in the everyday spoken language that describes it very well so that one can understand where we're going from here…It is true that this video here combines that, it gives you a picture of what is going on and how the research wants to proceed in a certain way and it has it very nicely written graphically, it just obviously needs other information about maybe where this is going. It shows in here that it will be reused for example in the process. What does reuse mean? Why on the one hand you say I want to have a low carbon footprint and on the other hand you store it and recycle depending on circumstances? What does that mean? Perhaps the person who hears and sees it gets confused?”.*
After watching the video, [S] brought up the fact that these technologies are very complicated, and the explanation needed to be simplified for her to have an opinion on whether she supports or not CCUS technologies. The rest of the participants agreed with her, and [B] mentioned that the societal context was missing from the video. There was a common agreement that the video presented CCUS as an already existing and developed technology, but in reality, it is not and there yet and there are still many unknowns in the CCUS process.

The participants expressed that better and more educational information was needed, but they differed in terms of who should be responsible for this. One participant suggested that communication about the project should come from the industrial partner involved in the project and the company should start by involving their employees and their families,

[J]: “ *I think we first start from the company itself or the companies that are creating the problem inside outside of quotes let’s say and create the production of let's say carbon dioxide and have a large human resource within the organisation. That workforce means that one employee is at least one family, and we are not even multiplying it. So, I would say that if you put our own region of, where we are, which is a partner and has almost 300 employees, that's a small community, since it's basically a thousand. It's a thousand people who from the company itself can be involved in the whole process. So, I think that the company itself through its own people should be the first to start any information and any dissemination of knowledge, so what is going on? what is it? How, are we going to deal with it? what is coming? what are the initiatives that other countries are taking let's say? What is Europe doing?*”.

Another participant suggested that should not be done only by the industrial partner, but there should also be an independent body.

[M]: *“…what is certain is that it cannot only come from the companies involved. It should also come from someone independent who will also tell them the opposite point of view. Because surely everyone will say what is in their own interest. What I think is a bit subtle is that in this case, let's say, to say to society that it's very important to reduce carbon dioxide emissions and for that, we're going to do, we're going to use some new technologies. You should tell them <laughs wryly> that there is a problem with the carbon dioxide being released, which they may not even be aware of”.*

This opinion was echoed by a participant who suggested that scientists might take on the role of communicating more unbiased information about CCUS.

[O]: *“The one who has the qualifications, the one who yes, maybe not so much communication skills. That's why I'd be pretty buttoned up myself. I agree with you on what you found. That gives it to a company that may know the techniques to approach, but there's a reservation people have towards that. I would agree that a scientist, a research centre, comes in and is more not neutral, and unbiased and that it doesn't lean on vested interests, possibly”.*

As demonstrated above, beliefs on the role of technology are not clear cut, as important information on the proposed technologies were absent from the communication video.

## Discussion

The findings from our study indicate that how CCUS was understood and perceived was influenced by interrelated factors related to local knowledge and concerns, societal context and understanding of CCUS technologies. Although we delineated these dimensions, they were all interrelated. There were many similarities between the mainland and island communities, but how they made sense of CCUS was also shaped by the particular local context. One interesting finding was that although the island community were at a potential storage site, they did not display greater concerns with the CCUS technology. In fact, it was the mainland community who were at the potential capture site that identified several potentially adverse implications of the technology for their area. This is in contrast with much of the literature that considers storage to be the most controversial element of the CCUS technology { Huijts *et al.,* 2007 #17513}{ Schumann *et al.,* 2014 #17514}{ Williams *et al.,* 2021 #17515}{ Arning *et al.,* 2020 #17516}. Given the explorative nature of this study, it is necessary to be careful about making too generalised conclusions based on this finding, but it should perhaps open up for further explorations of how people are impacted by CCUS across all of the sites that are implicated in the implementation of the technology.

All participants but one knew little about CCUS before the interviews. This aligns with past research that indicates general low public awareness of CCUS technologies ( Boyd *et al.*, 2017; Li *et al.*, 2014; Perdan *et al.*, 2017; Whitmarsh *et al.*, 2019). However, we found that when the participants were presented with even very limited information about CCUS they were able to relate it to their own situated knowledge and critically identify both local and wider potential issues with the technology. This resonates with previous studies on climate mitigation and adaptation projects that have shown how people can use their situated knowledge to critically assess new technologies regardless of complexity. Furthermore, the use of local knowledge can also help ensure social and environmentally better outcomes for these projects ( McNamara & Buggy, 2017; Rojas Blanco, 2006).

The societal context, including the history of experiences with companies and the local, national, and supranational political systems, shaped the participants' perception of new technological initiatives in the area. For some participants those past experiences were associated with deception and misinformation, often leading them to be sceptical about social acceptance as they were concerned about the information sources as well as the companies’ motivations. This aligns with extant research that indicates the important role past experiences play in shaping future perceptions and acceptance of new technological developments ( Holzinger *et al.*, 2011; Sulaymani *et al.*, 2022). Someone’s trust can be determined by their past experiences { Schwerter & Zimmermann, 2020 #17451. Trust between local communities and stakeholders that develop a project, is an important contextual factor for an operational and successful energy project { Walker *et al.*, 2010 #17452}{ Liu *et al.*, 2020 #17453}. Based on previous experiences, some of the participants expressed their discontent with previous industrial activities in the region, and that could impact their trust and support for new technological installations, such as the carbon capture facility. Notably, participants from the island mentioned the past activities of the “ *Ore Extraction Limited*” despite being 200 km away, and their operations not directly impacting them. 

On an abstract level participant were mostly positively inclined towards the role that CCUS could play in addressing climate change. However, when it came to a more specific and in-depth analysis, they raised several questions and concerns, they were uncertain about the exact implications the technologies would have in terms of resource usage, transport, and local economy. As discussed in the methods section, water issues were not mentioned in the communication video, so the researcher added that extra element to the discussion. This allowed for a co-construction of knowledge between the researcher and the participants, with the former using their local knowledge to contextualise CCUS within their community. Furthermore, participants from both communities were not certain that the technologies would be implemented in a transparent and beneficial way that would benefit the local communities. Predominantly the islanders had questions about local benefits from the storage facilities, and that was partially based on previous bad experiences with oil and gas companies.

This concern about the exact implications of a CCUS project also stemmed from the limited information they had received. In many cases, participants could not form an opinion about CCUS as the information they had was very limited and did not answer their questions. They discussed how some of the information communicated through the video was confusing and, in some cases, misleading. For them to be able to take decisions they wanted to have concrete data that were specific to their location. According to some of the participants, the video presented CCUS technologies as something that is well established, and that scientists are aware of all limitations, but that generated scepticism as to why despite the limitations those technologies were still pursued.

Aligning with participants’ perceptions, several researchers have indicated the uncertainties and limitations associated with CCUS technologies ( Beddies, 2015; Boyd, 2016; Lane *et al.*, 2021; Lee *et al.*, 2019). Pertinent literature suggests that when risks and uncertainties are not communicated towards the public, then the public becomes more sceptical of a project ( Ashworth *et al.*, 2012), supporting the value of two-way communication when it comes to the development of a carbon capture project ( Gough & Mander, 2014).

In Greece, we found that apart from the information and the communication that we provided as researchers, there was no other information or communication from any of the industrial partners or the project managers towards the local community. Especially within the island community, the storage potential of the area was unknown, despite the oil and gas company that holds the extraction rights of the area having made this information public on their website and other media outlets. In addition, some participants reported that the information provided through the video was lacking substance and not adequate to address their questions. The lack of transparency can have a negative impact on the social acceptance of carbon capture projects and often lead to the cancellation of such projects ( Beddies, 2015; Brunsting *et al.*, 2011; Oltra *et al.*, 2012). This presented a dilemma in the conduct of this study as there was confidential information regarding the project that we were not at liberty to relay that information to the public, inhibiting fully transparent discussion.

In addition to transparency, concerns over the use of tax-payers money were raised when the role of the EU was discussed. Similarly, to other studies, participants were concerned with potential environmental, health and societal negative impacts associated with CCUS. There was some uncertainty on how and why private companies were financed by tax-payers money to help with finding a solution to a problem that could potentially have serious implications for their ability to operate and be profitable.

### Implications, limitations, and future research

Despite the explorative nature of this study, our findings contribute to expanding understanding of public perceptions of CCUS in Greece and similar contexts, enabling policymakers, organisations and institutions to better engage and involve the local communities in future energy projects ( Kurath & Gisler, 2009; Perlaviciute & Squintani, 2020). As this study explores an emerging debate within the decarbonisation literature, we seek to publish these early results to make a timely contribution. Our future research will expand on these preliminary findings as local implementation of a CCUS demonstration facility continues. Due to the lack of available information and the novelty of the technologies, future research should consider more comprehensive information and dissemination methods to maximise peoples’ understanding of CCUS and their implications. As an example, one of the researchers during the interviews brought up the utilisation of water during the capture process and that might have framed CCUS in a negative manner ( de Vries *et al.*, 2016; Druckman & Bolsen, 2011). Adding to the above, there is also a time constraint associated with the interviews and the opinions participants form on a new subject can be limited. As discussed by Jones *et al.* (2017), more time and more information could alter peoples’ opinions on new technologies. Finally, this study has not explored the involvement of communities during an environmental permitting process, and such research would be important to be considered in the future.

## Conclusions

The aim of this study was to explore local perceptions about CCUS in a Greek national context to broaden our understanding of the dynamics that shape awareness and acceptance of CCUS. We explored two separate CCUS processes, that of carbon capture and that of carbon storage in two different communities. This preliminary study shows the importance of examining CCUS and social acceptance in relation to the specific social context of a project and looking at CCUS as an ecosystem rather than as an individual technologies and process. The dynamics that applied to this case study in two rural locations in Greece with previous experiences and controversies around extraction cannot be directly translated to other social contexts. What it is possible to say is that a place-based approach that can take account of social relationships and dynamics is more likely to give a detailed understanding of the factors that shape people’s perception of new technologies in that specific location.

Our findings suggest that despite the low awareness of CCUS amongst the participants, with limited information they were able to critically assess the technology and envision what potential environmental, economic, and social impacts it would have in the local area. Participants expressed some scepticism towards how the technology would be implemented and this was at times enhanced by the social and historical context of the area. Furthermore, the lack of detailed information meant that the participants did not feel they had adequate information to take a stance on any future CCUS project in the two areas. In our case, participants were asked to discuss something they had little or no knowledge of, apart from one participant who was well-informed about CCUS because of his professional expertise. Although some information was related to them via the video, that was not adequate for them to form an educated opinion. That brings to the forefront the importance of communication and education that should be taking place within these communities during such projects. Community members should be involved in the process from early on and help shape the project, rather than being asked their opinion after decisions have been made.

Furthermore, this study also indicates that local communities can play an important role in enabling a deeper understanding of the impact CCUS technologies might have in the local area by using their detailed local knowledge to identify and potentially tackle important issues that might only otherwise become evident later in any CCUS project. However, to do that in a more meaningful way there would need to be more comprehensive educational and communicational provisions that can give the communities the capabilities to understand and shape CCUS projects. Based on the findings of this study, CCUS technologies are a complex system of technologies and processes that sometimes the general public is not able to comprehend. Thus, local communities should be actively involved in the communication and education outreach programs aiming to simplify CCUS for a non-expert audience. Whilst it has been suggested that lack of knowledge is a limitation to public participation in decision-making ( Wang *et al.*, 2019), we argue that lack of transparency, and lack of collaboration from organisations, limit public engagement in energy-related projects.

## Data Availability

Despite our efforts to anonymise the interviews, due to the small geographical location where the data were collected, sharing the whole transcripts of the interviews could result in the identification of some of the participants.

## Footnotes

^1^ It is important to note that the company is registered with the EU ETS. EU ETS refers to an EU-wide mechanism that monitors and trades carbon emissions that are a result of industrial activities such as aviation, power stations, oil refineries, cement factories and oil and steel production. The EU ETS operates under a “cap and trade” foundation, where an allowance limit is set on greenhouse emissions per industrial company, and this maximum allowance is then converted to trading emission allowances ( Commission, 2021; Teixidó *et al.*, 2019). If companies exceed their emissions allowance, then they can buy additional allowances at a cost that is determined by the carbon market. To abate those emissions and avoid paying for extra allowances, the company needs to find solutions to reduce their carbon emissions, and CCUS is part of that effort.

^2^ https://www.youtube.com/watch?v=Ao5gI1ZNlic&t=9s
