# Supplementary material for: Public perception and acceptance of CCUS: preliminary findings of a qualitative case study in Greece
Source: Open Res Eur. 2024 Jul 24;3:205. Originally published 2023 Nov 21. [Version 3] doi: 10.12688/openreseurope.16663.3 (PMC11273079; doi:10.12688/openreseurope.16663.3)
Supplement: Peer review history

# Reviewer report: Reviewer response for version 3

**DOI:** 10.21956/openreseurope.19593.r42425

**Published:** 2024-07-25

**Authors:** Paul Upham

**Affiliations:** 1 IREES, ESRIG, University of Groningen, Groningen, Groningen, The Netherlands

I think this version is much improved and recommend for approval without reservation. The inclusion of additional data from the focus group, and the more tentative claims, help the paper alot.

Is the case presented with sufficient detail to be useful for teaching or other practitioners?

Yes

Is the work clearly and accurately presented and does it cite the current literature?

Yes

If applicable, is the statistical analysis and its interpretation appropriate?

Not applicable

Are all the source data underlying the results available to ensure full reproducibility?

Yes

Are the conclusions drawn adequately supported by the results?

Yes

Is the background of the case’s history and progression described in sufficient detail?

Yes

Reviewer Expertise:

Social acceptance of low carbon energy technology, including CCS.

I confirm that I have read this submission and believe that I have an appropriate level of expertise to confirm that it is of an acceptable scientific standard.

# Reviewer report: Reviewer response for version 2

**DOI:** 10.21956/openreseurope.18665.r39966

**Published:** 2024-05-15

**Authors:** Farah Mulyasari

**Affiliations:** 1 Pertamina University, Jakarta, Special Capital Region of Jakarta, Indonesia

It is a pleasure to read a social acceptance study of CCS/CCUS from different region in Europe. The research methods that span from data collection and analysis are sufficient. However, few suggestions might help in shaping the manuscript to a robust conclusion. For example, clear justification and criteria is needed for the sum and type of interviewee; a table/graph is advisable to pinpoint the key results from different themes (knowledge and concerns, societal context, etc.) hence the readers could better relate. In the conclusions part, specifically when talked about suggestions, it is better to clarify the examples of what is "more comprehensive educational and communicational provisions" be like.

Is the case presented with sufficient detail to be useful for teaching or other practitioners?

Yes

Is the work clearly and accurately presented and does it cite the current literature?

Partly

If applicable, is the statistical analysis and its interpretation appropriate?

I cannot comment. A qualified statistician is required.

Are all the source data underlying the results available to ensure full reproducibility?

Yes

Are the conclusions drawn adequately supported by the results?

Partly

Is the background of the case’s history and progression described in sufficient detail?

Yes

Reviewer Expertise:

disaster risk communication, public engagement for CCUS, climate change communication

I confirm that I have read this submission and believe that I have an appropriate level of expertise to confirm that it is of an acceptable scientific standard, however I have significant reservations, as outlined above.

## References

[1] : Potentials of the public engagement strategy for public acceptance and social license to operate: Case study of Carbon Capture, Utilisation, and Storage Gundih Pilot Project in Indonesia. *International Journal of Greenhouse Gas Control* .2021;108: 10.1016/j.ijggc.2021.103312 10.1016/j.ijggc.2021.103312

# Response

**Published:** 2024-07-02

**Authors:** KOSTAS STAVRIANAKIS

**Affiliations:** Robert Gordon University, UK

Dear reviewers, Thank you for your feedback and your input in strengthening our paper. Please see below the revisions we made to this paper after considering your feedback. The most significant addition we have included in this revised version is data from a focus group that we conducted in a potential nearby storage site. Initially we did not include that set of data, as we were planning to use them as part of another publication, but after the reviewers' suggestions we decided to include them in this manuscript. This allowed us to reframe the paper and explore a wider aspect of CCUS technologies, including the storage component rather than just the capture as we had before. As suggested by one of the reviewers, in line 300 we have added a table summarizing the findings and compares and contrasts the two different communities that we explored. One of the reviewers wanted more clarification on the “more comprehensive educational and communicational provisions” in the conclusion. We have addressed that by adding more information in lines 711-714 The title has been adjusted to better reflect the exploratory nature of this paper. We have also adjusted our methods, results and discussion to incorporate the findings from the focus group. Thank you

# Reviewer report: Reviewer response for version 2

**DOI:** 10.21956/openreseurope.18665.r38149

**Published:** 2024-05-09

**Authors:** Paul Upham

**Affiliations:** 1 IREES, ESRIG, University of Groningen, Groningen, Groningen, The Netherlands

I'm not sure what standards are expected here. This paper is fairly well-contextualised, but at this point the data and analysis remain light. I see it as a useful exploratory study.

Is the case presented with sufficient detail to be useful for teaching or other practitioners?

Yes

Is the work clearly and accurately presented and does it cite the current literature?

Yes

If applicable, is the statistical analysis and its interpretation appropriate?

Not applicable

Are all the source data underlying the results available to ensure full reproducibility?

Yes

Are the conclusions drawn adequately supported by the results?

Yes

Is the background of the case’s history and progression described in sufficient detail?

Yes

Reviewer Expertise:

Social acceptance of low carbon energy technology, including CCS.

I confirm that I have read this submission and believe that I have an appropriate level of expertise to confirm that it is of an acceptable scientific standard, however I have significant reservations, as outlined above.

# Reviewer report: Reviewer response for version 2

**DOI:** 10.21956/openreseurope.18665.r38151

**Published:** 2024-04-22

**Authors:** Christian Oltra

**Affiliations:** 1 Centro Investigaciones Energéticas, Medioambientales y Tecnológicas, Madrid, Spain

I overall think that the lack of a clear research question, the lack of a clear linkage between the collected data and the predefined aspects of the study, the limited number of interviews, the lack of a proper interview protocol and the lack of focus in the discussion undermines the significance of the article.

Is the case presented with sufficient detail to be useful for teaching or other practitioners?

Partly

Is the work clearly and accurately presented and does it cite the current literature?

Partly

If applicable, is the statistical analysis and its interpretation appropriate?

Not applicable

Are all the source data underlying the results available to ensure full reproducibility?

Partly

Are the conclusions drawn adequately supported by the results?

Partly

Is the background of the case’s history and progression described in sufficient detail?

Partly

Reviewer Expertise:

Risk perception; public attitudes towards energy technology; environmental attitudes

I confirm that I have read this submission and believe that I have an appropriate level of expertise to state that I do not consider it to be of an acceptable scientific standard, for reasons outlined above.

# Reviewer report: Reviewer response for version 1

**DOI:** 10.21956/openreseurope.17994.r36356

**Published:** 2024-01-02

**Authors:** Dimitrios Xenias

**Affiliations:** 1 Cardiff University, Cardiff, Wales, UK

**Public perception and acceptance of CCUS: preliminary findings of a qualitative case study in Greece**

 Thank you for this well-written paper, which I read with interest. The authors explored public perception / acceptance of a novel CCUS scheme in a mining/industrial community in Greece. This was part of a wider EU funded research programme.

 It is right and important to explore such topic qualitatively and in depth, as they are usually novel and unprecedented in the areas and communities involved. So it is useful to explore the understanding of such innovations at a local community level, as well as the meanings attributed to each scheme, and any anticipated impacts and expectations from it.

 However, doing so on the basis of just five interviews sounds quite minimal and I would hesitate to put much faith on results that are based in such small sample. Although this is a very good preliminary exploratory study, to be followed by a larger sample or replication, I find it too small to be published as a standalone paper as it stands. Does this journal support the publication of small pilot studies?

 Could this research be followed up with further interviews? Larger sample? Focus group discussion or similar? Or if the above are not possible, then other types of research and analysis that might complement the picture and add to our understanding of the local history, context and community as well as the company, its activities, and industrial relations. For example, there is mention of past promises and events within the same region with the same industry, and this might need to be exposed further for the benefit of this paper.

 Nonetheless, sample size remains my main concern. Perhaps, if there is no reem for further sample, replication or another complimentary study, a combination with other samples of this EU project might help bolster the conclusions of this paper? E.g. if other partners of this EU project ran similar community studies elsewhere, then their results could be compared and synthesised?

 To more specific comments:

 p.3 I would be cautious to describe CCUS, CCS, Wind, Solar and others collectively as ‘decarbonisation technologies’. Yes they all broadly contribute to decarbonisation targets, but I see a fundamental difference between a system that does not produce any CO2, to a system that actively sucks CO2 out of the atmosphere’ and is normally part of a nearby larger industrial complex.

 P.5 The authors mention that “although the themes are presented below in an individual basis, the intersections amongst them are the novelty of this paper.” Please can you clarify what this means?

 P.8  The authors discuss the role of contextual factors and I observed a notable exception from the list: the role of trust. This is a common finding in this kind of research and although it was only mentioned once in the summary of Theme 2, therefore I wonder whether the authors were not aware of this or did not think it was important to discuss.

Is the case presented with sufficient detail to be useful for teaching or other practitioners?

Yes

Is the work clearly and accurately presented and does it cite the current literature?

Yes

If applicable, is the statistical analysis and its interpretation appropriate?

Not applicable

Are all the source data underlying the results available to ensure full reproducibility?

No

Are the conclusions drawn adequately supported by the results?

Partly

Is the background of the case’s history and progression described in sufficient detail?

Yes

Reviewer Expertise:

Environmental Psychology, Climate Change, Behaviour Change

I confirm that I have read this submission and believe that I have an appropriate level of expertise to confirm that it is of an acceptable scientific standard, however I have significant reservations, as outlined above.

# Response

**Published:** 2024-02-02

**Authors:** KOSTAS STAVRIANAKIS

**Affiliations:** Robert Gordon University, UK

Dear reviewer,  Thank you for your helpful and constructive comments and helping us strengthen our paper. Please see below our response to your comments. The responses are indicated in the line numbers provided below and can be seen in purple text within the updated version.

 Reviewer 3: Approved with reservations

 Thank you for this well-written paper, which I read with interest. The authors explored public perception / acceptance of a novel CCUS scheme in a mining/industrial community in Greece. This was part of a wider EU funded research programme.

 It is right and important to explore such topic qualitatively and in depth, as they are usually novel and unprecedented in the areas and communities involved. So, it is useful to explore the understanding of such innovations at a local community level, as well as the meanings attributed to each scheme, and any anticipated impacts and expectations from it.

 However, doing so on the basis of just five interviews sounds quite minimal and I would hesitate to put much faith on results that are based in such small sample. Although this is a very good preliminary exploratory study, to be followed by a larger sample or replication, I find it too small to be published as a standalone paper as it stands. Does this journal support the publication of small pilot studies? Could this research be followed up with further interviews? Larger sample? Focus group discussion or similar? Or if the above are not possible, then other types of research and analysis that might complement the picture and add to our understanding of the local history, context and community as well as the company, its activities, and industrial relations. For example, there is mention of past promises and events within the same region with the same industry, and this might need to be exposed further for the benefit of this paper.

 Nonetheless, sample size remains my main concern.

**Reply: **As we say on lines 55-58 this is a preliminary case study from an early report from a larger international project. Regarding sample size we have strengthened out justification of interviews in lines 160-165. Whilst the method of the study is replicable, the purpose of this study is not to be replicated in the sense that you can generalise the findings if you repeat the study. As discussed on the paper, opinions and perceptions can change depending on different contextual factors, thus expecting to replicate the study with similar results would not be constructive. We have discussed in the manuscript the notion of representation in qualitative studies and included citations exemplifying the importance of the small n number in qualitative research. Perhaps, if there is no reem for further sample, replication or another complimentary study, a combination with other samples of this EU project might help bolster the conclusions of this paper? E.g. if other partners of this EU project ran similar community studies elsewhere, then their results could be compared and synthesised?

   To more specific comments:

 p.3 I would be cautious to describe CCUS, CCS, Wind, Solar and others collectively as ‘decarbonisation technologies. Yes, they all broadly contribute to decarbonisation targets, but I see a fundamental difference between a system that does not produce any CO2, to a system that actively sucks CO2 out of the atmosphere’ and is normally part of a nearby larger industrial complex.

**Reply: **This is an interesting point, and this has been changed to “technologies” in line 72 and rephrased.

 P.5 The authors mention that “although the themes are presented below in an individual basis, the intersections amongst them are the novelty of this paper.” Please can you clarify what this means?

**Reply: **We have added further consideration of the thematic intersectionality. Please refer to lines 208-213 and line 438.

 P.8  The authors discuss the role of contextual factors, and I observed a notable exception from the list: the role of trust. This is a common finding in this kind of research and although it was only mentioned once in the summary of Theme 2, therefore I wonder whether the authors were not aware of this or did not think it was important to discuss.

**Reply:** As also per the suggestion of reviewer 2 we have expanded on the role of trust. Please refer to line 457-464.

# Reviewer report: Reviewer response for version 1

**DOI:** 10.21956/openreseurope.17994.r36354

**Published:** 2024-01-02

**Authors:** Christian Oltra

**Affiliations:** 1 Centro Investigaciones Energéticas, Medioambientales y Tecnológicas, Madrid, Spain

Introduction

 The literature review on public perceptions and acceptance of Carbon Capture and Storage (CCS) appears limited, lacking depth and breadth. The research justification is weak, with an unclearly articulated problem statement. Moreover, the absence of a defined research question and specific objectives further dilutes the study's foundation. The rationale for conducting research on social acceptance in the context of the CCUS project is not clear, especially given its design - a pilot plant adjacent to an existing industrial plant for partial carbon dioxide capture. The justification for selecting Greece as a unique case study for understanding the interplay between CCUS implementation and social acceptance is not convincingly argued.

 Method

 The methodological approach, particularly the use of a case study research design as per Yin (2009), is inadequately connected to this study's specific design. Furthermore, the limited number of interviews conducted raises questions about the comprehensiveness of the findings. The ambiguity surrounding the profile of the interviewees, whether they are residents or stakeholders and their sociodemographic details, further weakens the methodological robustness.

 Results

 The analysis of the data seems disjointed from the study's analytical dimensions. There is a lack of clear linkage between the collected data and the predefined aspects of the study, which hampers the effectiveness of the results section.

 Discussion

 While the discussion presents some interesting ideas, the overall lack of focus in the article undermines the significance of this section. An example of this is the reference to previous research on the impact of past experiences on perceptions and acceptance of new technologies. This idea, although relevant, is not sufficiently developed or integrated into the broader narrative of the study, resulting in a discussion that lacks relevance and depth.

Is the case presented with sufficient detail to be useful for teaching or other practitioners?

Partly

Is the work clearly and accurately presented and does it cite the current literature?

Partly

If applicable, is the statistical analysis and its interpretation appropriate?

Not applicable

Are all the source data underlying the results available to ensure full reproducibility?

Partly

Are the conclusions drawn adequately supported by the results?

Partly

Is the background of the case’s history and progression described in sufficient detail?

Partly

Reviewer Expertise:

Risk perception; public attitudes towards energy technology; environmental attitudes

I confirm that I have read this submission and believe that I have an appropriate level of expertise to state that I do not consider it to be of an acceptable scientific standard, for reasons outlined above.

# Response

**Published:** 2024-02-02

**Authors:** KOSTAS STAVRIANAKIS

**Affiliations:** Robert Gordon University, UK

Dear reviewer, Thank you for your helpful and constructive comments and helping us strengthen our paper. Please see below our response to your comments. The responses are indicated in the line numbers provided below and can be seen in purple text within the provided document.  

 Reviewer 2: Not approved.

 Introduction

 The literature review on public perceptions and acceptance of Carbon Capture and Storage (CCS) appears limited, lacking depth and breadth. The research justification is weak, with an unclearly articulated problem statement. Moreover, the absence of a defined research question and specific objectives further dilutes the study's foundation. The rationale for conducting research on social acceptance in the context of the CCUS project is not clear, especially given its design - a pilot plant adjacent to an existing industrial plant for partial carbon dioxide capture. The justification for selecting Greece as a unique case study for understanding the interplay between CCUS implementation and social acceptance is not convincingly argued.

**Reply: **In lines 31-44 we discuss the importance of examining social acceptance of CCUS in a local context and argue that most previous studies have been in Northern Europe or North America. The rationale and research scope of the study can be seen in lines 54-62 and the justification for Greece is explained in lines 63-83.

 Method

 The methodological approach, particularly the use of a case study research design as per Yin (2009), is inadequately connected to this study's specific design. Furthermore, the limited number of interviews conducted raises questions about the comprehensiveness of the findings.  The ambiguity surrounding the profile of the interviewees, whether they are residents or stakeholders and their sociodemographic details, further weakens the methodological robustness.

**Reply:** Whilst we do not agree with the critique on our methods, we have added more information in lines 151-154 and 160-164 for the case study design and lines 203-206 for participants’ characteristics. We have discussed in the manuscript the notion of representation in qualitative studies and included citations exemplifying the importance of the small n number in qualitative research.

 Results

 The analysis of the data seems disjointed from the study's analytical dimensions. There is a lack of clear linkage between the collected data and the predefined aspects of the study, which hampers the effectiveness of the results section.

**Reply: **We are confident that the analysis of the data aligns with the purpose of the study. Discussion

 While the discussion presents some interesting ideas, the overall lack of focus in the article undermines the significance of this section. An example of this is the reference to previous research on the impact of past experiences on perceptions and acceptance of new technologies. This idea, although relevant, is not sufficiently developed or integrated into the broader narrative of the study, resulting in a discussion that lacks relevance and depth.

**Reply:** As also per the suggestion of reviewer 3, we have expanded on the role of trust and previous experiences. Please see lines 457-464.

# Reviewer report: Reviewer response for version 1

**DOI:** 10.21956/openreseurope.17994.r36355

**Published:** 2023-11-24

**Authors:** Paul Upham

**Affiliations:** 1 IREES, ESRIG, University of Groningen, Groningen, Groningen, The Netherlands

This paper describes the background, methods and results of a small number of interviews with members of the public living close to a prospective site that will capture and store CCS for reuse (CCUS).

 The authors need to provide more information on exactly what the interviewees were told - e.g. via a link to the video referred to and via appending other information conveyed. The authors also need to provide more information on the proposed CCUS development - is carbon storage to be onsite, in what form, how transported out etc.

 The paper is generally well-written, analysed and executed. However the number of interviewees seems very small and the research design justification relating to this is under developed. Did I understand correctly that 5 people were interviewed once each? Or were they each interviewed multiple times? Please clarify this. Either way, I think you also need supplementary justification. If you interviewed each individual multiple times, then this is a longitudinal form of interviewing that would add strength to the empirical part of the case, and more should be made of this.

 If you only interviewed 5 people once, then you again need to strengthen the justification for this. You can (i) estimate the fraction of the village that this constitutes; (ii) you can and should give us a little more demographic information on the interviewees, so we know a little about them; (iii) you can and should make a case for in-depth, semi-ethnographic work in which the researcher has embedded themselves in the community (as you seem to have done); (iv) for the benefit of people concerned about representativeness, you can explain a little more about the value of small n qualitative work.

 One other thing: theme 1 on Knowledge: although knowledge was involved, I read these comments as being also, or even primarily, about a concern with impacts.

Is the case presented with sufficient detail to be useful for teaching or other practitioners?

Yes

Is the work clearly and accurately presented and does it cite the current literature?

Yes

If applicable, is the statistical analysis and its interpretation appropriate?

Not applicable

Are all the source data underlying the results available to ensure full reproducibility?

Yes

Are the conclusions drawn adequately supported by the results?

Yes

Is the background of the case’s history and progression described in sufficient detail?

Yes

Reviewer Expertise:

Social acceptance of low carbon energy technology, including CCS.

I confirm that I have read this submission and believe that I have an appropriate level of expertise to confirm that it is of an acceptable scientific standard, however I have significant reservations, as outlined above.

# Response

**Published:** 2024-02-02

**Authors:** KOSTAS STAVRIANAKIS

**Affiliations:** Robert Gordon University, UK

Thank you for your helpful and constructive comments and helping us strengthen our paper. Please see below our response to your comments. The responses are indicated in the line numbers provided below and can be seen in purple text within the provided document.  

 Reviewer 1: Approved with reservations.

 This paper describes the background, methods and results of a small number of interviews with members of the public living close to a prospective site that will capture and store CCS for reuse (CCUS).

 The authors need to provide more information on exactly what the interviewees were told - e.g. via a link to the video referred to and via appending other information conveyed. The authors also need to provide more information on the proposed CCUS development - is carbon storage to be onsite, in what form, how transported out etc.

**Reply:** We have added further information on the pilot project in line 108-112. There is a link to the video in the manuscript. We have also added the YouTube link as a footnote in line 172.

 The paper is generally well-written, analysed and executed. However, the number of interviewees seems very small and the research design justification relating to this is underdeveloped. Did I understand correctly that 5 people were interviewed once each? Or were they each interviewed multiple times? Please clarify this. Either way, I think you also need supplementary justification. If you interviewed each individual multiple times, then this is a longitudinal form of interviewing that would add strength to the empirical part of the case, and more should be made of this.

**Reply:** Six people were interviewed, and each person was interviewed once. We have adjusted the writing in the manuscript in lines 180 and 185

 If you only interviewed 5 people once, then you again need to strengthen the justification for this. You can (i) estimate the fraction of the village that this constitutes; (ii) you can and should give us a little more demographic information on the interviewees, so we know a little about them; (iii) you can and should make a case for in-depth, semi-ethnographic work in which the researcher has embedded themselves in the community (as you seem to have done); (iv) for the benefit of people concerned about representativeness, you can explain a little more about the value of small n qualitative work.

**Reply: **We have added some participants’ characteristics in lines 203-206 and included more context on our semi-ethnographic approach in lines 151-154. We have added some more explanation on the purpose of this case study, and why it is not about representation in lines 160-165. We have discussed in the manuscript the notion of representation in qualitative studies and included citations exemplifying the importance of the small n number in qualitative research.

 One other thing: theme 1 on Knowledge: although knowledge was involved, I read these comments as being also, or even primarily, about a concern with impacts.

**Reply: **This is a helpful observation and we have  amended the name of theme from “Knowledge” to “Knowledge and concerns”.
